# Impact of COVID-19 Pandemic on Radiology Department Employees and Trainees in Al-Qassim, 2021

**DOI:** 10.7759/cureus.57294

**Published:** 2024-03-30

**Authors:** Ali Albweady, Maryiah AlHajji, Rawaf AlBassam, Hala Almalki, Beshair Almansour, Renad Alghofaili, Manal Alsubaie

**Affiliations:** 1 Department of Radiology, College of Medicine, Qassim University, Al-Mulaida, SAU; 2 Department of Radiology, King Fahad Medical City, Riyadh, SAU

**Keywords:** radiology, trainees, employees, radiology department, covid-19

## Abstract

Objectives

This study aimed to assess the impact of the COVID-19 pandemic on radiology department employees and trainees. It also compared the impact of COVID-19 to the pre-COVID-19 era in the Al-Qassim region.

Methods

This was a quantitative observational analytical cross-sectional study conducted in the largest government hospitals under the Ministry of Health (MOH) in Al-Qassim. A pre-determined questionnaire was distributed among radiology staff that included demographic characteristics, the impact of the COVID-19 pandemic among radiology staff, the behavior of staff related to COVID-19 infection, and the assessment of mental health using the patient health questionnaire (PHQ-9).

Results

Eighty-four radiology staff were recruited (64.3% males vs 35.7% females). Of these, 66.7% were trainees and the rest were employees (33.3%). Of the trainees, 32.1% and 42.9% thought that elective imaging, procedures, and outpatient/clinic exposures were reduced during the pandemic, and 37.5% indicated that their training had been affected negatively. The prevalence of depression among radiology staff was 36.9%. The prevalence of depression was substantially higher among radiology trainees (p=0.038), those who were not infected with COVID-16 (p=0.041), and those who indicated that their studying time increased at the time of the pandemic (p=0.047). However, after conducting multivariate regression analysis, these variables did not seem to have significantly affected depression (p>0.05).

Conclusion

Training and medical education have been affected negatively because of the outbreak. Studying time and research activities of employees and trainees slowed down, which could be critical to their careers. Trainees complained about the significant reduction in their exposure to clinics and imaging procedures. Therefore, a method to safeguard the well-being of employees and trainees in the radiology department is necessary to limit the impact of such pandemics.

## Introduction

By January 2020, 41 cases of pneumonia with an unknown etiology were documented in Wuhan, Hubei Province, China. Subsequently, this was announced as a new form of coronavirus, temporarily labeled as the 2019 new coronavirus (2019-nCoV), responsible for the outbreak in 2019, as recognized by the World Health Organization (WHO) [1]. The outcome of this pandemic was intense, affecting various fields, including global economies, lifestyles, and healthcare systems. Consequently, the WHO officially classified coronavirus disease 2019 (COVID-19) as a pandemic [2]. As a result of the pandemic, healthcare settings worldwide were customized to adapt to new criteria of health management aimed at enhancing capacity.

A study conducted in the United Kingdom (UK) revealed that all the activities were suspended, with a reconfiguration of staff duties and responsibilities across various locations and new situations, focusing primarily on urgent care. Additionally, practice in the radiology department was significantly reduced in activity due to the negative impact of COVID-19 [3]. Another study showed a decrease exceeding 50% in radiology practice, particularly evident in the use of mammography and elective ultrasound, which dropped by 80% [4]. Additionally, the impact was particularly notable in outpatient imaging services. Due to the disruption caused by COVID-19, a reassessment of financial reserves and academic practices may be needed to include elements to mitigate risk in the system. As a result of this impact, remote work has increased, which has resulted in increased job satisfaction, reduced stress levels, and improved turnaround times [4].

Another study that was conducted to evaluate the impact of this pandemic indicated that clinical radiography employees are key patient-facing personnel responsible for the handling of most diagnostic imaging procedures and the provision of routine radiotherapy treatments [5]. Additionally, due to the acute phase of the epidemic in the UK, the National Cancer Research Institute (NCRI) implemented major adaptations in the delivery of radiation services. As surgical capacity decreased during the pandemic, there was an increase in the use of radiotherapy as the first definitive cure for cancer [5]. 

According to research conducted to examine the influence of COVID-19, radiology training came to a total halt during this period. All future tests were rescheduled, rotations were halted, and trainees were redeployed to intensive care units, emergency rooms, and medical wards. This disruption had a particularly negative impact on practical training in modalities including ultrasound, fluoroscopy, and interventional radiology. Additionally, the growing rates of staff illness posed another challenge. Symptomatic staff members, or those living with symptomatic individuals, needed time off for self-isolation. During this period, there was no infrastructure in place to facilitate remote access to reporting, engaging in virtual tutorials, or attendance at multidisciplinary meetings (MDMs). Consequently, this group of trainees had limited real-time interaction with the specialty. To address this issue, more regular programs have been reintroduced, and measures have been taken to address the backlog. Shift patterns have been adjusted to allow extra time slots, ensuring that trainees spend more time delivering acute care and reducing supervised training sessions, which resulted in a lack of training depth and breadth [6].

On March 2, 2020, the first confirmed case of COVID-19 in Saudi Arabia was reported in the eastern region [7]. Since then, infection prevention and control measures expanded throughout the region. Specifically, the Saudi Central Board for Accreditation has modified its essential standard parameters for Middle East respiratory syndrome (MERS) to also include COVID-19 [8]. Additionally, the Saudi Ministry of Health advised delaying all non-urgent screening tests and treatments until the condition was stabilized. Cases were asked to be rescheduled in accordance with hospital management directives. Outpatient radiological imaging and procedures were prioritized for acute conditions and cases where imaging could change patient management. Imaging of patients with COVID-19, whether suspected or confirmed, was done in the event of a changing circumstance or if the patient's management needed to be adjusted. Non-urgent imaging in patients known or suspected to have developed COVID-19 was postponed until they were deemed non-contagious [9]. 

A study investigating the influence of the COVID-19 pandemic on residency and fellowship training programs in Saudi Arabia revealed that healthcare systems had to instantly reorganize their operations to address the emergency, aiming to optimize resources while mitigating the spread of the outbreak. One of the most immediate changes observed in educational programs was the widespread cancellation of in-person medical meetings and seminars, which were replaced with video seminars, live streaming, or webinars. The predominant mode of communication between residents, fellows, and tutors had been regular simulated learning; the increased utilization of telematics education services tried to bridge the training gap. Considering the extremely contagious nature of COVID-19 as well as other emerging infections, face-to-face interactions in large-group contexts (such as lectures) could have potentially served as hotspots for disease transmission. In response, several residency programs established team-based structures where clinical responsibilities and tasks were delegated to a group of residents, while others served as standby teams. Other challenges and aspects of such crises, such as the impact on the mental health of both healthcare workers and patients, were unfortunately disregarded or overlooked. Mental diseases, behavioral changes, and emotional trauma are all believed to be caused by catastrophic occurrences. Trainee residents and hospital-based fellows who were in close contact with suspected or confirmed cases of COVID-19 were at an increased risk of contracting the disease. This had a substantial effect on their mental health for various reasons [10].

## Materials and methods

This study used a cross-sectional quantitative observational analytical approach and was conducted in the largest government hospitals under the Ministry of Health (MOH) in the Al-Qassim region. The study was approved by the Ministry of Health, Saudi Arabia (approval number: H-04-Q-001).

Participants were selected as a representative sample from each hospital using a simple random selection approach. The study included employees and trainees from the radiology department who agreed to participate. Individuals who refused to participate, were unavailable during data collection, or did not complete the questionnaire were excluded. The sample size was calculated using Epi Info™ Version 6 (Centers for Disease Control and Prevention, Atlanta, Georgia, United States) with a population size (N) of 78, determined using the Kish formula for sample size estimation [7].

A pre-determined questionnaire was used which included questions on demographic characteristics, the impact of the COVID-19 pandemic among radiology staff, staff behavior related to the COVID-19 infection, and the assessment of mental health using the patient health questionnaire (PHQ-9).

Data were presented using numbers, percentages, mean, and standard deviation whenever appropriate. The level of depression was compared with the basic demographic characteristics of participants by using the Fischer Exact test. Significant results achieved were then placed in a multivariate regression model to determine the significant independent risk factor for depression, with corresponding odds ratio as well as 95% confidence interval being reported. A P-value <0.05 was considered statistically significant. All data analyses were performed using IBM SPSS Statistics for Windows, Version 21.0 (Released 2012; IBM Corp., Armonk, New York, United States).

## Results

The number of radiology staff members who responded to our survey was 84. The socio-demographic characteristics of the participants are shown in Table 1. The majority of the respondents were from Buraidah Central Hospital (20.2%), with around two-thirds (66.7%) being trainees and 39.3% being technologists. Males accounted for 64.3% of the respondents, while females comprised 35.7%. Respondents living with their families constitute 72.3%. When asked about the overall departmental workload during this pandemic, 28.6% indicated a reduction, while 27.4% indicated it was the same as before.

**Table 1:** Demographic characteristics of employees and trainees in the radiology department (N = 84)

Study variables	n (%)
Hospital	
King Fahd Specialist Hospital	16 (19.0%)
Buraidah Central Hospital	17 (20.2%)
Maternity and Children Hospital, Buraidah	9 (10.7%)
King Saud Hospital, Unaizah	7 (8.3%)
Al Rass General Hospital	7 (8.3%)
Albukairyah General Hospital	8 (9.5%)
Almidhnab General Hospital	9 (10.7%)
Riyadh Alkhabra General Hospital	2 (2.4%)
Albadayea General Hospital	9 (10.7%)
Radiology staff category	
Employees	28 (33.3%)
Trainees	56 (66.7%)
Profession	
Resident physician	16 (19.0%)
Consultant	8 (9.5%)
Technologist	33 (39.3%)
Technician	18 (21.4%)
Nurse	2 (2.4%)
Specialist	7 (8.3%)
Gender	
Male	54 (64.3%)
Female	30 (35.7%)
Marital status	
Single	33 (39.3%)
Married	51 (60.7%)
Living with	
Alone	17 (20.2%)
Family	61 (72.3%)
Colleagues/friends	6 (7.1%)
During this pandemic, the overall departmental workload has	
Significantly reduced	11 (13.1%)
Reduced	24 (28.6%)
Same as before	23 (27.4%)
Increased	22 (26.2%)
Significantly increased	4 (4.8%)

Table 2 illustrates the impact of the COVID-19 pandemic on radiology trainees. It is notable that 32.1% of the trainees perceived a reduction in elective imaging and procedure exposure during the pandemic. Additionally, 42.9% indicated a decrease in outpatient/clinic exposure, while 39.3% observed an increase in on-call/emergency room (ER) exposure during this crisis. Similarly, 37.5% of the trainees indicated that their training had been negatively affected by COVID-19. Trainees indicated that the hospitals to which they are currently assigned for rotation had partially ceased operations. The percentage of trainees who had outside rotations that had been canceled due to COVID-19 was 55.4%, while the percentage of trainees whose elective rotations were canceled due to the pandemic was 46.4%.

**Table 2:** Impact of COVID-19 pandemic among radiology trainees (N = 56) COVID-19: coronavirus disease 2019

During this pandemic	n (%)
Your elective imaging and procedure exposure (such as mammography and elective outpatient ultrasound)	
Significantly reduced	08 (14.3%)
Reduced	18 (32.1%)
Same as before	16 (28.6%)
Increased	13 (23.2%)
Significantly increased	01 (01.8%)
Your outpatient/clinic exposure	
Significantly reduced	05 (08.9%)
Reduced	24 (42.9%)
Same as before	15 (26.8%)
Increased	10 (17.9%)
Significantly increased	02 (03.6%)
Your on-call or ER patient exposure	
Significantly reduced	05 (08.9%)
Reduced	10 (17.9%)
Same as before	12 (21.4%)
Increased	22 (39.3%)
Significantly increased	07 (12.5%)
How much has your training been affected by COVID-19?	
Significantly affected in a negative way	09 (16.1%)
Affected in a negative way	21 (37.5%)
Has not been affected	12 (21.4%)
Affected in a positive way	09 (16.1%)
Significantly affected in a positive way	05 (08.9%)
The hospital or hospitals you were rotating at, ceased operations	
Entirely	08 (14.3%)
Partially	26 (46.4%)
Did not cease any operations	22 (39.3%)
Did you have an outside rotation (away rotation) that got canceled because of COVID-19?	
Yes	31 (55.4%)
No	25 (44.6%)
Did you have elective rotations that got canceled because of COVID-19?	
Yes	26 (46.4%)
No	30 (53.6%)

In Table 3, it is shown that 67.9% of respondents had examined a positive COVID-19 patient. While 64.3% indicated that there was an adequate supply of personal protective equipment (PPE) at their current workplace. Nearly 60% of the respondents indicated that their hospital provided immediate guidelines or protocols during the COVID-19 pandemic. Furthermore, a great proportion (76.2%) of radiology department staff consistently practiced hand hygiene (regular hand washing). Approximately 38% of the respondents indicated that their program director regularly monitored their condition. Additionally, 41.7% of respondents indicated being recruited to cover other staff. The prevalence of respondents who tested positive for COVID-19 infection was 26.2%.

**Table 3:** Behavior of radiology employees and trainees in the COVID-19 pandemic and infection (N = 84) COVID-19: coronavirus disease 2019; PPE: personal protective equipment

Variables	n (%)
Have you examined a positive COVID-19 patient?	
Yes	57 (67.9%)
No	27 (32.1%)
Did you have enough PPE at your hospital?	
Yes	54 (64.3%)
No	07 (08.3%)
Partially	23 (27.4%)
Did your hospital provide guidelines or protocols about outpatient visits and procedures during the COVID-19 pandemic?	
Yes, immediately	50 (59.5%)
Yes, gradually, with the development of the epidemic	27 (32.1%)
No	07 (08.3%)
Have you been washing your hands?	
Always	64 (76.2%)
Usually	15 (17.9%)
Sometimes	05 (06.0%)
Has your program director been asking about you and checking your conditions?	
Yes, always	32 (38.1%)
Yes, very often	15 (17.9%)
Yes, sometimes	18 (21.4%)
Yes, rarely	04 (04.8%)
No	15 (17.9%)
Have you been recruited for the coverage of COVID-19 patients? (like for shortage coverage in the ICU)	
Yes	35 (41.7%)
No	49 (58.3%)
Have you been tested for COVID-19?	
No	30 (35.7%)
Yes, and the result is negative	30 (35.7%)
Yes, and it’s pending	02 (02.4%)
Yes, and it’s positive	22 (26.2%)

**Figure 1:**
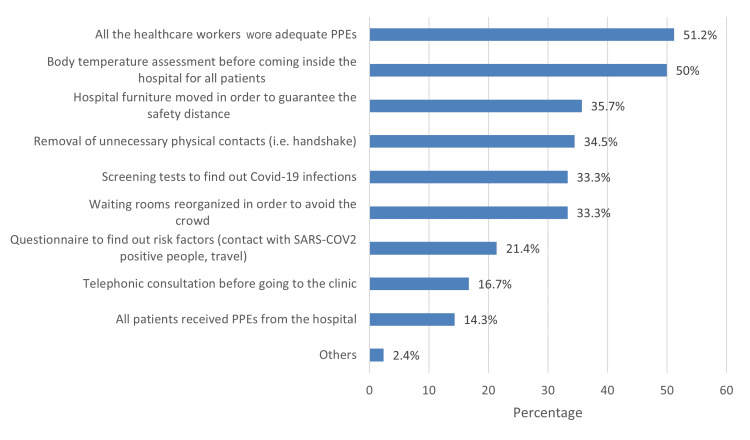
Adapted precautions to limit the transmission of the infection during the outpatient visits PPE: personal protective equipment; SARS-COV2: severe acute respiratory syndrome coronavirus 2; Covid-19: coronavirus disease 2019

The most commonly implemented precaution to limit the transmission of infection during outpatient visits was ensuring that all healthcare workers wear appropriate PPE (51.2%), followed by conducting body temperature assessments for all patients before entering the hospital (50%) (Figure 1).

**Figure 2:**
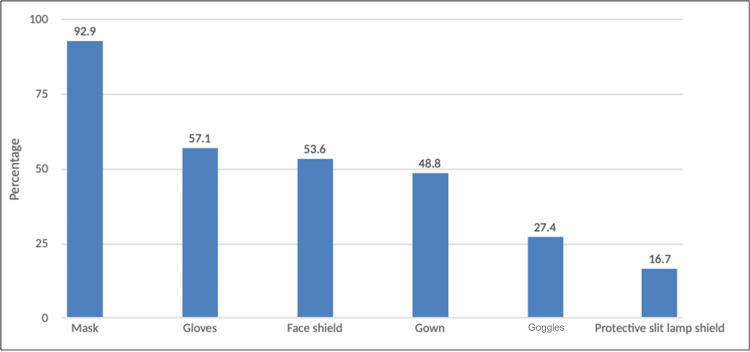
Types of adapted protective measures

The most common type of protective measure during the COVID-19 pandemic was wearing a mask (92.9%), followed by gloves (57.1%), and a face shield (53.6%) (Figure 2).

Table 4 presents the characteristics of respondents who were infected by COVID-19. Among them, 45.5% indicated contracting the disease from the patient. The prevalence of infected staff experiencing any symptoms was 77.3%, with the most common symptoms being fever (45.5%), cough (36.4%), and loss of smell (36.4%). The percentage of staff who had been hospitalized due to the COVID-19 infection was 27.3%, with the most commonly administered treatments being supportive care (33.3%) and hydroxychloroquine (33.3%).

**Table 4:** Characteristics of employees and trainees who were diagnosed with COVID-19 infection (n = 22) * Variable with multiple responses.

Variables	n (%)
From whom had you contracted the infection?	
Patient	10 (45.5%)
Colleague	02 (09.1%)
Family member or relative	05 (22.7%)
Friends	03 (13.6%)
I don’t know	02 (09.1%)
Did you have any symptoms?	
Yes	17 (77.3%)
No	05 (22.7%)
Experienced symptoms of COVID-19 *	
Fever	10 (45.5%)
Cough	08 (36.4%)
Loss of smell	08 (36.4%)
Shortness of breath	04 (18.2%)
Diarrhea	04 (18.2%)
Malaise	03 (13.6%)
Sore throat	03 (13.6%)
Have you been hospitalized before?	
Yes	06 (27.3%)
No	16 (72.7%)
If yes, what sort of treatment did you receive? (n = 6)	
Supportive	02 (33.3%)
Mechanical ventilation	01 (16.7%)
Hydroxychloroquine	02 (33.3%)
Dexamethasone	01 (16.7%)

Of the respondents, 58.3% reported that their current program transitioned to an internet-based modality due to COVID-19, with webinars being the most common type of online modality (61.2%) and Zoom applications (Zoom Video Communications, Inc., San Jose, California, United States) being the most commonly used web applications (81.6%). When asked to rate their satisfaction with virtual webinars, virtual presentations, and virtual exams or tests, 21.4%, 15.5%, and 15.5% rated very satisfactory. Additionally, the proportion of respondents who believed that after the pandemic all theoretical education in radiology residencies will not be conducted via web-based platforms was assessed.

**Table 5:** Teachings and meetings during the COVID-19 pandemic (N = 84) COVID-19: coronavirus disease 2019

Variables	n (%)
Has your program started any internet-based education modalities because of the COVID-19 pandemic?	
Yes	49 (58.3%)
No	35 (41.7%)
Types of online modalities that have been participated in or attended * ^†^	
Webinars	30 (61.2%)
Presentations	16 (32.7%)
Exams or tests	7 (14.3%)
Video consultations on real patients	4 (8.2%)
None	6 (12.2%)
Type of web app used for those meetings ^†^	
Zoom (Zoom Video Communications, Inc., San Jose, California, United States)	40 (81.6%)
Microsoft Teams (Microsoft Corporation, Redmond, Washington, United States)	5 (10.2%)
Adobe Connect (Adobe Inc., San Jose, California, United States)	2 (4.1%)
Google Hangouts (Google LLC, Mountain View, California, United States)	2 (4.1%)
On a scale of 1 to 5 (where 1 is very unsatisfactory and 5 is very satisfactory) please rate the meetings organized by Saudi Radiology programs and the Saudi Radiology Society:	
Rate Virtual Webinars	
Very satisfactory	6 (7.3%)
Unsatisfactory	6 (7.1%)
Neutral	17 (20.2%)
Satisfactory	16 (19.0%)
Very satisfactory	18 (21.4%)
I did not attend any	21 (25.0%)
Rate Virtual Presentation	
Very satisfactory	6 (7.1%)
Unsatisfactory	3 (3.6%)
Neutral	18 (21.4%)
Satisfactory	21 (25.0%)
Very satisfactory	13 (15.5%)
I did not attend any	23 (27.4%)
Rate Virtual Exams and Tests	
Very satisfactory	2 (2.4%)
Unsatisfactory	5 (6.0%)
Neutral	16 (19.0%)
Satisfactory	17 (20.2%)
Very satisfactory	13 (15.5%)
I did not attend any	31 (36.9%)
Do you believe that after the pandemic is over, all the theoretical education in radiology residencies should be done using web-based platforms?	
Yes	35 (41.7%)
No	24 (28.6%)
I don’t know	25 (29.8%)

Of the respondents, 34.5% and 40.5% indicated that their studying time and their research activities were the same as before the pandemic (Table 6).

**Table 6:** Research activities during the COVID-19 pandemic (N = 84) COVID-19: coronavirus disease 2019

Variables	n (%)
Research, studying, and electives: during this pandemic, your studying time	
Significantly reduced	4 (4.8%)
Reduced	26 (31.0%)
Same as before	29 (34.5%)
Increased	22 (26.2%)
Significantly increased	3 (3.6%)
During this pandemic, your research activity has	
Significantly reduced	12 (14.3%)
Reduced	24 (28.6%)
Same as before	34 (40.5%)
Increased	14 (16.7%)
Significantly increased	0

The descriptive statistics of the PHQ-9 questionnaire are presented in Table 7. It was observed that the mean score of PHQ-9 was 7.61 (SD 7.61), with 36.9% of respondents classified as depressed and 63.1% as not depressed. Regarding the severity of depression, minimal, mild, moderate, moderately severe, and severe depression were observed among 42.9%, 23.8%, 22.6%, 8.3%, and 2.4% of the respondents, respectively.

**Table 7:** Descriptive statistics of PHQ-9 questionnaire (n = 84) PHQ-9: patient health questionnaire

Variables	N (%)
PHQ-9 total score (mean ± SD)	7.61 ± 5.99
Level of depression	
Depressed (≥10 points)	31 (36.9%)
Not depressed (<10 points)	53 (63.1%)
Severity of depression	
Minimal	36 (42.9%)
Mild	20 (23.8%)
Moderate	19 (22.6%)
Moderately severe	07 (08.3%)
Severe	02 (02.4%)

The prevalence of depression was observed to be more common among radiology trainees (p = 0.038), those who were not diagnosed with COVID-19 infection (p = 0.041), and those who perceived an increase in study time during the pandemic (p = 0.047) (Table 8). However, no significant relationship was observed between depression levels and profession, gender, marital status, living status, the overall workload of the department, being recruited for the coverage of COVID-19 patients, examining COVID-19 patients, or engaging in activities that did not show a significant relationship with the level of depression (p > 0.05).

**Table 8:** Impact of the COVID-19 pandemic on the mental health of radiology department staff (N = 84) § The P-value has been calculated using the Fischer exact test. ** Significant at the p<0.05 level.

Factor	Level of depression		P-value ^§^
	Depressed (n = 31), n (%)	Not Depressed (n = 53), n (%)	
Radiology staff category			
Employees	6 (19.4%)	22 (41.5%)	0.038 **
Trainees	25 (80.6%)	31 (58.5%)	
Profession			
Resident physician	7 (22.6%)	9 (17.0%)	0.750
Consultant	2 (06.5%)	6 (11.3%)	
Technologist	14 (45.2%)	19 (35.8%)	
Technician	6 (19.4%)	12 (22.6%)	
Nurse/Specialist	2 (6.5%)	7 (13.2%)	
Gender			
Male	21 (67.7%)	33 (62.3%)	0.645
Female	10 (32.3%)	20 (37.7%)	
Marital status			
Single	15 (48.4%)	18 (34.0%)	0.248
Married	16 (51.6%)	35 (66.0%)	
Living with			
Alone	6 (19.4%)	11 (20.8%)	0.143
Family	25 (80.6%)	36 (67.9%)	
Colleagues/friends	0	6 (11.3%)	
During this pandemic, the overall departmental workload has			
Reduced	15 (48.4%)	20 (37.7%)	0.602
Same as before	7 (22.6%)	16 (30.2%)	
Increased	9 (29.0%)	17 (32.1%)	
Have you examined a positive COVID-19 patient?			
Yes	23 (74.2%)	34 (64.2%)	0.468
No	8 (25.8%)	19 (35.8%)	
Have you been recruited for the coverage of COVID-19 patients?			
Yes	12 (38.7%)	23 (43.4%)	0.819
No	19 (61.3%)	30 (56.6%)	
Diagnosed with COVID-19			
Yes	4 (12.9%)	18 (34.0%)	0.041 **
No	27 (87.1%)	35 (66.0%)	
Research, Studying, and Electives: During this pandemic, your studying time			
Reduced	10 (32.3%)	20 (37.7%)	0.047 **
Same as before	7 (22.6%)	22 (41.5%)	
Increased	14 (45.2%)	11 (20.8%)	
During this pandemic, your research activity			
Reduced	15 (48.4%)	21 (39.6%)	0.690
Same as before	11 (35.5%)	23 (43.4%)	
Increased	5 (16.1%)	9 (17.0%)	

Based on the significant results, a multivariate regression analysis was subsequently performed in Table 9 to determine the significant independent predictor of depression. Accordingly, we found that radiology staff diagnosed with COVID-19 and studying time during the pandemic did not show a significant effect on depression after adjustment to a regression model (all p>0.05).

**Table 9:** Multivariate regression analysis to determine the significant independent risk factors for depression (N=84) AOR: adjusted odds ratio; CI: confidence interval

Factor	AOR	95% CI	P-value
Radiology staff category			
Employees	Ref		
Trainees	2.094	0.672 – 6.526	0.203
Diagnosed with COVID-19			
Yes	0.456	0.125 – 1.661	0.234
No	Ref		
Research, studying, and electives: during this pandemic, your studying time			
Reduced	Ref		
Same as before	2.231	0.717 – 6.945	0.166
Increased	3.200	0.962 – 10.641	0.058

## Discussion

The present study aims to evaluate the impact of the COVID-19 pandemic on radiology employees and trainees in Al-Qassim Hospitals, Saudi Arabia. In efforts to limit the transmission of the virus, various safety measures were implemented through the outbreak, including social distancing at workplaces, the imposition of curfews, the suspension of international and domestic flights, and the implementation of remote learning [11]. One significant change in training and education programs was the widespread cancellation of face-to-face meetings and conferences. These were primarily replaced by webinars or recorded lectures. These measures were necessary to control the outbreak and reduce the risk of exposure. In this study, approximately 58.3% of the respondents started online-based education mainly through webinars (61.2%) conducted via Zoom meetings (81.6%). However, many of the staff expressed their satisfaction or high satisfaction with the outcomes of virtual webinars, presentations, and examinations, with 41.7% indicating a preference to continue online education for the theoretical part even after the pandemic. Alahmadi et al. also reported satisfaction with using webinars [12]. In their study, it was observed that 55.4% of the ophthalmology residents were satisfied with the virtual method of education, with Zoom being the main application used in the meetings (78.4%). Similarly, in Madinah, Saudi Arabia, 45% of diagnostic radiology trainees reported a shift towards web-based case-based educational activities, with directives to use online question banks and lectures (44%), while 22% indicated that case-based activities were on hold, and only 11% reported no change [13]. In Canada, a considerable percentage (91.7%) of radiology residents reported that virtual teaching rounds, change in schedules (78.1%), and online/phone readouts (72.9%) were the most utilized methods in Canadian radiology residency programs during the pandemic, with reported satisfaction levels ranging from neutral to higher levels [13]. In the United Kingdom, data indicates an overall improvement in attendance, volume, and training quality due to the increased accessibility through remote learning methods [14]. However, it was emphasized that in-person training is important for practical skills. Additionally, the Royal College of Radiologists' Junior Radiologists' forum webinars were well-accepted by all trainees, with a continuation of the series recommended, achieving a 54% satisfaction rate with the remote reporting available.

Approximately 41.7% of the respondents indicated that the overall workload of the department was significantly reduced or decreased during the pandemic, with a similar impact observed on their study time and research activities. This contrasts with the findings of Akudjedu et al., who observed an increased workload, particularly in COVID-19 cases, at the onset of outbreaks, although reduced work patterns were noted among non-COVID-19 cases [5]. In a study conducted by Coppola et al., over 60% of respondents estimated a workload increase of greater than 50%, with 40% expressing moderate to severe concern regarding the potential negative impact on their professional activities, including training and education [15]. This concern may potentially affect the management of non-COVID-19 patients, given the expected workload following the pandemic.

Many of the radiology trainees voiced concerns about a significant reduction in exposure to imaging procedures and clinical rotations, coupled with an increase in ER patients during the pandemic. Similarly, a great proportion of trainees stated a cancellation of their rotations outside their workplace (55.4%) or their elective rotations (46.4%). Consequently, their training and education were negatively affected. This finding aligns with studies conducted in Dammam, Saudi Arabia [10], where residents and fellowship trainees reported a significant decrease in training activities, corroborating similar findings in Riyadh, Saudi Arabia [12]. In Madinah, Saudi Arabia, radiology diagnostic trainees reported a moderate to severe impact, as well as a minimal to moderate negative impact, on educational and clinical activities [11]. It was concluded that certain novel methods and techniques, such as online lectures and presentations, had been implemented by training programs and the Saudi Council for Health Specialties (SCFHS) to reduce the detrimental effects on trainees. Employees and trainees who had direct contact with COVID-19 patients were susceptible to infection, as evidenced by 26.2% of radiology staff testing positive for COVID-19, with 45.5% reporting transmission through patient contact. This is consistent with the findings of Balhareth et al., who reported that 43% of the respondents had direct contact with COVID-19 patients, with 29% subsequently contracting the disease [10]. In a study published by Alahmadi and colleagues, only 4.2% of ophthalmology residents tested positive for COVID-19, with one resident being hospitalized and two receiving supportive treatment [12]. It was noted that their program directors always monitored their health conditions, aligning with our findings.

The psychological impact of the COVID-19 pandemic was also addressed in this study. More than one-third (36.9%) of the radiology departmental staff experienced depression, ranging from mild (23.8%) to moderate (22.6%), moderately severe (8.3%), and severe depression (2.4%). Furthermore, radiology trainees who complained of an increase in study time had been greatly affected by this mental condition. However, this effect did not reach statistical significance after adjustments to a predictive model (p>0.05). Hence, further investigations are required to validate these associations. In a study by Alahmadi et al., a considerable percentage of the residents (70.5%) reported mental health disorders during the pandemic, with 33.1% and 26.1% having mild to moderate depressive symptoms [12]. Other studies have reported an impact on anxiety or stress levels [10,13]. Depression, anxiety, and frustration at varying levels were observed among healthcare workers in their efforts to combat outbreaks [16]. Uncertainty remains a constant characteristic of the COVID-19 pandemic [10] and requires effective communication to manage the evolving dynamics of the virus. However, the relationship between experienced uncertainties and risk communication efforts was complicated by the surge of misinformation during the outbreak [17].

The generalization of this study was bound to some limitations. First, our sample size was relatively small (N=84), making it difficult to detect significant differences between the groups. Also, being a cross-sectional survey is prone to bias and does not measure cause and effect.

## Conclusions

The COVID-19 pandemic had a lot of impact on various domains of the Al-Qassim Hospital radiology department. Training and medical education were negatively affected by the outbreak. The studying time and research activities of both employees and trainees slowed down, posing potential challenges to their career advancement. Trainees reported a notable reduction in their exposure to clinics and imaging procedures. Furthermore, the pandemic had a considerable effect on the psychological states of employees and trainees. Further, regardless of the effect, a significant burden of depression was observed among trainees, particularly those who reported an increase in their studying time during this crisis. The mental well-being of healthcare staff should be monitored, with access to psychological support available within the hospital institution. Additionally, virtual learning is vital as an alternative educational approach during such events. To ensure the continuity of high-quality education, institutions should be flexible and capable of adapting to changes, with frequent curriculum revisions and evaluations, even during times of crisis. Finally, implementing methods to safeguard the well-being of employees and trainees in the radiology department is necessary to reduce the impact of such events.
